# Decline in activities of daily living in the rarer dementias

**DOI:** 10.1136/gpsych-2024-101905

**Published:** 2025-06-08

**Authors:** Beatrice Taylor, Suraya Mohamud, Emilie Brotherhood, Emma Harding, Claire Waddington, Paul M Camic, Daniel Alexander, Sebastian Crutch, Joshua Stott, Chris Hardy, Neil P Oxtoby

**Affiliations:** 1 Centre for Medical Image Computing, University College London, London, UK; 2 Dementia Research Centre, Queen Square Institute of Neurology, University College London, London, UK; 3 Adapt Lab, Research Department of Clinical, Educational and Health Psychology, University College London, London, UK

**Keywords:** Patient Care

## Abstract

Rarer dementias are associated with atypical symptoms and younger onset, which result in a higher burden of care. We provide a review of the global literature on longitudinal decline in activities of daily living (ADLs) in dementias that account for less than 10% of dementia diagnoses. Published studies were identified through searches conducted in Medical Literature Analysis and Retrieval System Online (MEDLINE), Excerpta Medica Database (Embase), Excerpta Medica Care (Emcare), PsycINFO, and Cumulative Index in Nursing and Allied Health Literature (CINAHL). The search criteria included terms related to ‘rarer dementias’, ‘activities of daily living’ and ‘longitudinal or cross-sectional studies’ following a predefined protocol registered. Studies were screened, and those that met the criteria were citation searched. Quality assessments were performed, and relevant data were extracted. 20 articles were selected, of which 19 focused on dementias within the frontotemporal dementia/primary progressive aphasia spectrum, while one addressed posterior cortical atrophy. Four studies were cross-sectional and 16 studies were longitudinal, with a median duration of 2.2 years. The Disability Assessment for Dementia was used to measure decline in 8 of the 20 studies. The varied sequences of ADL decline reported in the literature reflect variation in diagnostic specificity between studies and within-syndrome heterogeneity. Most studies used Alzheimer’s disease staging scales to measure decline, which cannot capture variant-specific symptoms. To enhance care provision in dementia, ADL scales could be deployed postdiagnosis to aid treatment and planning. This necessitates staging scales that are variant-specific and span the disease course from diagnosis to end of life. PROSPERO registration number: CRD42021283302.

## Introduction

 Dementia is a clinical syndrome of progressive cognitive decline with impaired social or occupational functioning. Of the major neurodegenerative dementias, the most common cause is typical Alzheimer’s disease (tAD), which is estimated to account for 50%–75% of dementia diagnoses.^1^ tAD is sporadic, has an older age of onset of symptoms (>65 years) and is characterised by episodic memory loss.

**Figure 1:**
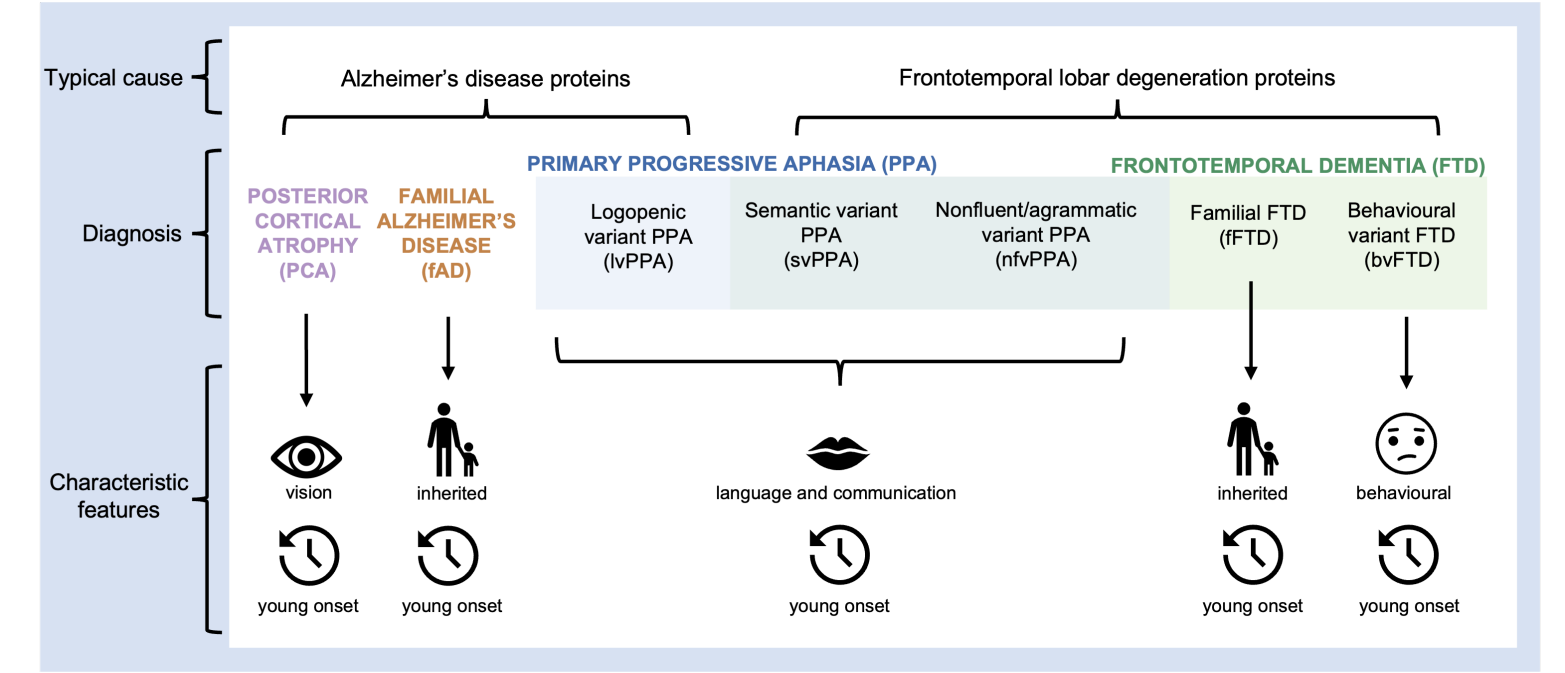
The typical causes and characteristic features of rarer dementias included in this review. Colour coding indicates the broader diagnostic categories and the overlapping categorisation of the primary progressive aphasias and the frontotemporal dementias.

This review considers ‘rarer dementias’, defined as those variants whose prevalence is estimated as less than 10% of total dementia diagnoses, and furthermore are neither sporadic amnestic AD presentation nor vascular aetiologies. These atypical dementias are associated with progressive difficulties with cognitive faculties other than memory, often develop at a relatively younger age (<65 years) and may be autosomal dominantly inherited.^2^ Of the named canonical dementia variants, the rarer dementias include: primary progressive aphasia (PPA), frontotemporal dementia (FTD), posterior cortical atrophy (PCA) and familial AD (fAD). Figure 1 illustrates the rarer dementias considered in this review and their primary pathologies, though there are exceptions to these clinicopathological associations.^3^

FTD is an umbrella term describing clinical syndromes caused by progressive deterioration of networks centred on the frontal and temporal lobes. It is estimated to account for 2% of all dementia diagnoses.^1^ The term encompasses behavioural variant FTD (bvFTD),^4^ semantic variant PPA (svPPA) and non-fluent/agrammatic variant PPA (nfvPPA).^5^

PPA is an overlapping term with FTD that refers to dementias characterised by initial changes in speech and/or language.^6 7^ It is estimated to account for <1% of all dementia diagnoses^6^ (based on a prevalence of three cases per 100 000 dementia diagnoses observed in the UK and a UK dementia prevalence of 850 000/67.22 million). The three most common variants are svPPA, nfvPPA and logopenic variant PPA (lvPPA). While svPPA and nfvPPA are associated with frontotemporal lobar degeneration (FTLD), lvPPA is predominantly caused by AD pathology.^8^

PCA is characterised by changes in visuoperceptual and visuospatial processing.^9 10^ The most common pathology associated with PCA is AD,^11^ with an estimated 5% of patients with AD presenting with PCA in specialist cognitive clinics.^12^

fAD and familial FTD (fFTD) are dominantly inherited forms of AD and FTD, respectively, typically having a younger onset.^13 14^ The prevalence of fAD is estimated at less than 1% of all AD diagnoses,^15^ while approximately 30% of FTD cases have some familial history of the condition.^14^ Consequently, both conditions constitute less than 1% of all dementia diagnoses. While symptom presentation in these dementias is comparable to the sporadic variants, the younger onset and the fact that they are dominantly inherited result in specific care needs relating to the stress placed on familial caregivers, since they have a genetic susceptibility to develop the same condition.^16^

The term ‘activities of daily living’ (ADL) refers to functional acts that are necessary to live independently, encompassing both basic ADL (BADL) such as eating, washing and continence, as well as instrumental ADL (IADL) such as organising finances, correspondence and cooking. In tAD, staging scales have been developed that provide detailed descriptions of how these ADLs change over the course of the disease.

For people living with dementia, care needs can be complex and difficult to address, which reduces their and their carers’ health-related quality of life. In rarer dementias, this is exacerbated by atypical symptoms.^17 18^ The rarer dementias may also be classified as young-onset dementia (YOD), diagnosed before the age of 65. While dementia at any age is challenging, experiencing it at a younger age raises additional concerns and care needs related to factors such as employment, mortgages and young children. Furthermore, individuals with YOD often experience a significant delay to diagnosis, which results in difficulties accessing appropriate care at the right time.^19^

Studies on carers’ experiences in the rarer dementias have indicated a need for better education about the disease.^20 21^ Understanding the longitudinal decline in ADLs is crucial for enabling the anticipation of care needs, helping carers prepare emotionally and practically for each expected symptom. Staging scales are fundamental for evaluating novel care interventions and can be used as outcome measures in clinical trials.^22^

While there have been systematic reviews of ADL in the more prevalent dementias,^23^,^25^ there is limited research into ADLs in people with rarer dementias despite them representing 1 in 10 dementia diagnoses. This review addresses the question, ‘What do we know about how symptoms pertaining to ADL change and evolve over the course of the disease for people living with a rarer dementia?’

## Methods

### Protocol and registration

The protocol for this systematic review was prepared according to Preferred Reporting Items for Systematic Reviews and Meta-Analyses (PRISMA) guidelines,^26^ and registered with International Prospective Register of Systematic Reviews (PROSPERO) (registration: CRD42021283302) prior to data extraction.

### Search strategy and eligibility criteria

A search was conducted across the following five databases: Medical Literature Analysis and Retrieval System Online (MEDLINE), Excerpta Medica Database (Embase), Excerpta Medica Care (Emcare), PsycINFO, and Cumulative Index in Nursing and Allied Health Literature (CINAHL), from inception up to 7 September 2021. The search contained keywords related to longitudinal or cross-sectional studies AND keywords related to activities of daily living AND keywords related to rarer dementias. The full list of search terms is included in the online supplemental material.

Studies were included if they met the following:

1. Were specifically about a dementia whose prevalence was estimated to account for less than 10% of total dementia diagnoses. This included PPA, svPPA, lvPPA, nfvPPA, PCA, FTD, bvFTD, fFTD, fAD;
2. Primarily addressed the ADLs of the person with dementia. IADLs and BADLs were both considered;
3. Were either longitudinal (ie, reporting on the same individuals over time) or cross-sectional (ie, explicit comparison of individuals at different disease stages);
4. Had a sample size of at least five people living with a rarer dementia, analysed separately to control participants;
5. Were published in English in a peer-reviewed journal.

Studies were excluded if they were about tAD, vascular dementia, Lewy body dementia or non-neurodegenerative dementia.

### Screening procedure

A three-step screening procedure was used: first by title, then by abstract and finally, by reading the full text. All stages were conducted by the primary reviewer (BT). At all three stages, a random 10% of the articles were screened by an independent rater (SM). Disagreements were discussed by the reviewers (BT and SM) and resolved. The studies identified for inclusion were manually citation searched using Google Scholar in March 2022 (BT and CH) to identify other relevant studies.

### Data extraction

We used a spreadsheet to store extracted data in the following domains: author name(s), publication year, size of the sample, study design, length of follow-up, setting (eg, the clinic that subjects were recruited from), diagnostic criteria, genetic testing, use of *in vivo* measures to confirm diagnosis (eg, neuroimaging), confirmation of diagnosis at autopsy, what measurement was used for ADLs, method of analysis (eg, statistical assessment, linear regression), the reported sequence of ADL decline and the rate of ADL decline.

### Quality rating

A quality assessment of the included literature was undertaken independently by two reviewers (BT and CH) using an adapted version of the quality assessment tools from the Joanna Briggs Institute,^27^ selected because they have previously been employed in systematic reviews of care needs in dementia.^28^ Our adapted version allowed the inclusion of additional questions relevant to specific areas of the systematic review, for example, ‘Were ADLs measured in a valid and reliable way?’

## Results

### Selection processes

A total of 579 unique studies were retrieved. Of these, 33 were sought for retrieval based on title and abstract, 32 articles were retrievable and read in full by the reviewer and 14 were deemed eligible for inclusion. Citation searches of these 14 records identified a further 6 articles, resulting in a total of 20 articles. Online supplemental figure 1 shows the PRISMA diagram.

### Synthesis

We grouped studies according to the syndrome/disease under consideration. To facilitate comparison across dementia variants, we used prevailing contemporary diagnostic terminology throughout the study (eg, svPPA rather than semantic dementia). Online supplemental table 1 summarises the recorded diagnosis of the studies’ participants and the contemporary interpretation. Online supplemental table 2 shows the main characteristics and outcomes of interest for each included study. Where given in the original study, p values were included in the table. Due to the small amount of literature, the findings of all studies were reported, with results from higher-quality studies presented first according to the quality rating in online supplemental table 3.

### Study characteristics and participants

The 20 studies included a total of 3309 participants diagnosed with a rarer dementia, some of whom may have been included in multiple studies. Of these participants, the average age at the start of the study was 67.26 years. Study countries included Australia (k=4), Brazil (k=2), France (k=2), Italy (k=1), Japan (k=1), UK (k=4) and the USA (k=5). For one study, the country was unclear. Four studies were cross-sectional. The remaining 16 studies were longitudinal, of which 10 were case series studies and 6 were cohort studies. In the longitudinal studies, study duration ranged between 1 and 11 years, with a median of 2.2 years. Table 1 gives a breakdown of study time frames per dementia variant.

**Table 1:** Distribution of studies included in the review by dementia diagnosis versus time frame of study

Observation period	Rarer dementias						
	PCA	FTD	bvFTD	PPA	svPPA	lvPPA	nfvPPA
Cross-sectional	29 ●		37 ●		37 ●		38 ●
			31 ●		38 ●		
			38 ●				
1 year		48 ●	49 ●	49 ●	42 ●	34 ●	42 ●
			42 ●		39 ●		39 ●
			39 ●		35 ●		34 ●
							35 ●
1–3 years		40 ●		44 ●	41 ●	41 ●	41 ●
3–5 years			36 ●	30 ●	33 ●	33 ●	33 ●
					36 ●		
5–7 years		43 ●	32 ●				
>7 years				46 ●	47 ●		

Colours indicate what domain of ADLs was considered: BADLs only—blue; IADLs only—green; BADLs and IADLs—red.

Numbers in the table represent reference citations.

ADLs, activities of daily living; BADLs, basic activities of daily living; bvFTD, behavioural variant FTD; FTD, frontotemporal dementia; IADLs, instrumental activities of daily living; lvPPA, logopenic variant PPA; nfvPPA, non-fluent/agrammatic variant primary progressive aphasia; PCA, posterior cortical atrophy; PPA, primary progressive aphasia; svPPA, semantic variant primary progressive aphasia.

Despite the literature search encompassing a broad set of rarer dementia terminology, 19 of the 20 studies focused on people living with a dementia diagnosis in the least rare FTD/PPA spectrum. The remaining study focused on PCA.^29^ 10 studies compared multiple rarer dementias within the FTD/PPA spectrum. As expected for low-prevalence diseases, small sample sizes were observed, with just three studies exceeding 100 individuals in any single dementia variant.^30^,^32^

All cohorts studied were recruited through specialist memory clinics. Five studies were conducted using data collected from the FRONTIER Frontotemporal Dementia Group, Australia;^3133^,^36^ four were based in Addenbrookes Hospital, UK;^2937^,^39^ and three used data collected by the National Alzheimer’s Coordinating Center, USA.^30 32 40^ All studies used clinical diagnostic criteria to identify patients. Some studies also used neuroimaging techniques to confirm diagnosis, such as magnetic resonance imaging^3338 39 41^,^44^ or A*β* positron emission tomography (PET).^41 44^ One study confirmed pathology at autopsy.^40^

The most popular measure of ADL decline was Disability Assessment for Dementia (DAD),^45^ which was used in eight studies. The DAD was primarily developed to assess functional decline in tAD—it consists of 40 items, of which 17 address BADLs and 23 address IADLs. Some studies used custom informant questionnaires to measure ADLs that were tailored to specific dementia variants. One study developed and validated a new measure, the FTD Rating Scale (FRS).^39^ All but one study^43^ used statistical methods to describe longitudinal change. Significance tests were used to verify decline; regression was used to predict ADL decline.^32 33^ Some studies adjusted for covariates to statistically account for confounding factors.^31 32 44 46^ There was considerable variation in the description and assessment of ADL decline. Six studies only collected data at two time points, resulting in symptoms categorised as having already occurred at baseline or appearing after 12 months. The most detailed study sequentially ordered eight ADLs based on data collected over 10 years.^47^ Some studies found that the rate of decline was faster in rarer dementias compared with tAD.^34 42 48^ Two studies explicitly discussed the implications of the results for addressing care needs.^29 39^

### Results by dementia variant

#### Frontotemporal dementia

Three studies included people living with FTD without differentiating between variants.^40 43 48^ All studies were longitudinal, with a duration in the range of 1–6 years and a mean of 2.7 years. Annual rates of decline in ADLs found that bathing, grooming and toileting declined significantly faster in FTD than tAD.^40^ Dressing and eating were found to be the best-preserved ADLs.^43^ One study considered Pick’s disease (an older diagnostic term which is broadly synonymous with FTD) with 10 individuals having pathological confirmation and 34 showing ‘prominent language impairments and subtle personality changes’.^48^ ADL decline was reported to be significantly faster in Pick’s disease compared with tAD.^48^

#### Behavioural variant FTD

Eight studies included people living with bvFTD,^3132 36^,^39 42 49^ including one study of individuals living with frontal variant FTD.^37^ The duration of five longitudinal studies ranged from 1 to 6 years, with a mean of 2.4 years. Two studies identified increased appetite/overeating as an initial symptom in bvFTD.^36 37^ The sequence of ADL decline was found to be IADLs followed by BADLs,^42^ specifically: initiation > planning > BADLs.^38^ The abilities to use the stove and to travel were found to decline significantly faster in bvFTD than in tAD; however, the overall rate of functional decline was comparable.^32^ The rate of functional decline was found to be faster in bvFTD compared with svPPA or nfvPPA, using a single follow-up 12 months after baseline.^39^

#### Primary progressive aphasia

Four studies included people living with PPA without differentiating by variant.^30 44 46 49^ All studies were longitudinal, with durations in the range of 1–11 years and a mean of 4.75 years. One study found that a decline in toileting, hygiene and dressing occurred 5 years after onset, while eating and walking declined 7 years after onset.^46^ Another study reported the sequence of ADL decline as: verbal communication > transactions > meal preparation > self-care > ambulation.^30^ Using a different approach, one study^44^ differentiated participants by amyloid-beta status using florbetapir PET scans. This study reported the sequence of ADL decline in amyloid beta positive (A*β*+) individuals as: communication > employment and recreation >‘shopping and’ money > travel > household care > self-care. Among individuals who were amyloid beta negative (A*β*−), ‘shopping and money’ declined later.

#### Semantic variant primary progressive aphasia

Nine studies included people living with svPPA.^3335^,^39 41 42 47^ Among the seven longitudinal studies, the duration was in the range of 1–10 years, with a mean of 3 years. One study gave a particularly clear description of the sequence of ADL decline, reporting that symptoms occurred as: reading/writing > work/housework > daily activities > eating > continence > dressing.^47^ Another study found that the first symptom of functional decline was rigid routines and unusual food choices.^36^ Annual rate of decline in ADLs was found to be highest in planning, using a single follow-up 12 months after baseline.^42^

There was conflicting evidence as to whether the rate of IADL decline was more pronounced in nfvPPA^35^ or svPPA.^38^ The rate of functional decline was comparable between svPPA and tAD.^42^

#### Non-fluent/agrammatic variant primary progressive aphasia

Seven studies included people living with nfvPPA.^33^,^3538 39 41 42^ Among the six longitudinal studies, the duration was in the range of 1–3.5 years, with a mean of 1.7 years. The initial symptoms of functional decline were found to be language and household chores.^34^ There was variation as to whether the decline occurred concurrently in BADLs and IADLs^34 42^ or whether the decline in BADLs preceded the decline in IADLs.^38^

#### Logopenic variant PPA

Three studies included people living with lvPPA.^33 34 41^ All studies were longitudinal, with a duration in the range of 1–3.5 years and a mean of 2.3 years. Across the literature, this was the least represented of the three named PPA variants. Decline in IADLs was more notable than decline in BADLs, with a marked decline in planning and execution 12 months after baseline.^34^

#### Posterior cortical atrophy

One study included people living with PCA.^29^ This cross-sectional study reported that the sequence of ADL decline started with impairment in managing finances/correspondence and meal preparation, followed by impairment across BADLs.

### Study quality

The quality of the included studies was assessed using a quality assessment tool adapted from the Joanna Briggs Institute critical appraisal tools, with additional questions to address bias specific to the topic, as shown in online supplemental table 3. Appraisals specific to cohort, cross-sectional and case series study designs are given in online supplemental figures 4–6, respectively. No studies were excluded during the quality appraisal. All studies reported clear inclusion criteria, and most reported using objective, standard criteria for diagnosis. Demographics were clearly and appropriately reported by all studies, statistical analyses were uniformly appropriate and the conclusions of all studies were clearly supported by the data. For longitudinal studies, follow-up was often incomplete, reflecting high levels of drop-out associated with disease progression among participants.

## Discussion

We conducted a systematic review of longitudinal change in ADLs in people diagnosed with a rarer dementia. As expected, the sequences and rates of ADL decline in rarer dementias differed from tAD and differed between conditions. Even within a single dementia syndrome, there was variation in the reported sequence in which ADLs declined, as well as variation in the rate of decline, reflecting either study-level variation in diagnostic specificity or genuine within-syndrome heterogeneity. Each rarer dementia is itself an umbrella term which conceals variation in both pathology and disease presentation. Moreover, since the publication of the earliest study in this review in 1999, there have been advancements in disease definitions and diagnostic criteria, such as the addition of lvPPA to consensus criteria in 2011.^7^ There was a trend for more recent studies to integrate neuroimaging or genetic screening into their diagnosis and participant stratification. This could enhance comprehension of the heterogeneity in symptom presentation, as variations within clinical variants might correspond to differences in disease mechanisms or neuroanatomy.

Among the studies included in our review, only one fell outside of the FTD/PPA spectrum. This is consistent with prevalence estimates, as FTD is among the more common rarer dementias, accounting for approximately 2% of dementia diagnoses in the UK.^1^ Notably, there were no studies on early onset dementias and their additional age-related care needs.^21^ This suggests an acute need for descriptions of ADL decline in these often-familial conditions.

Most studies in this review relied on tAD staging scales to assess ADL decline, potentially leading to an under-reporting of variant-specific ADL. The only rarer dementia-specific staging scales of ADL decline referenced in the literature were the FRS^39^ and the FTLD-specific Clinical Dementia Rating,^50^ both of which assess FTD decline. While the FRS assesses a range of basic and IADLs, it lacks items specifically related to language, limiting its utility for tracking symptom severity in the PPAs. Newer tools such as the Progressive Aphasia Severity Scale^51^ or the symptom-led staging systems for PPA developed by Hardy and colleagues^52 53^ may be useful additional tools in this area.

To provide the best, personalised care in rarer dementias, we need to understand the sequence of functional decline, from initial presentation to end of life, in each variant. One way to do this is to focus on the development of variant-specific staging scales comparable to those used in tAD. Staging scales that provide detailed descriptions of functional decline can enable carers to make legal, financial, care planning and housing decisions, thus reducing carer burden^20 21^ and supporting independence for people living with dementia. The studies in this review failed to equally represent ADL in all (early, middle and late) stages of decline, thus limiting their translation into staging scales. Consideration should also be given to the relationship between specific cognitive processes and ADLs: some of the included studies on FTD, for instance, highlighted the role of executive function in relation to IADL,^3132 36^,^39 42 49^ so future research should elaborate on the specific linkages between neuropsychological profiles of these rarer dementias and their functional consequences.

Future work could use emerging research into wearable technology^54^ to gather data on a broader spectrum of ADLs tailored both to the specific type of dementia and even to the specific individual. This approach would enable the creation of data sets with richer, objective measures of ADLs and allow for more frequent and regular data collection. With larger data sets comes the possibility of leveraging statistical or computational models to gain deeper, quantitative insight into ADL decline.

### Limitations

This review has some limitations. We encompassed various types of dementia, which restricted the ability to directly compare studies. Additionally, the combination of cross-sectional and longitudinal studies prevented us from conducting a meta-analysis. Quality appraisal by two independent researchers strengthened the results. Nonetheless, some of the included studies had minor shortcomings (see online supplemental figures 3–4) that may have influenced the review’s outcomes. Issues such as incomplete follow-up in longitudinal studies could result in an incomplete depiction of decline over time, while insufficient demographic data hindered the assessment of participant representativeness.

## Conclusions

Our review highlights the heterogeneity of ADL decline in the rarer dementias. Our evidence supports the need for more comprehensive and variant-specific descriptions of longitudinal decline in ADL in rarer dementias, particularly outside the FTD/PPA spectrum. Indeed, studies frequently used measures developed for tAD that are not fit for purpose, given the atypical symptoms and younger onset that are characteristic of rarer dementias. Given the variability associated with these conditions, it could be particularly useful to use inductive or qualitative methods to allow for unanticipated insights to emerge, grounded in the lived experiences of people affected by these conditions, to make sure that the most meaningful milestones of impact are delineated and can be better supported in the future.^55^

## Supplementary material

10.1136/gpsych-2024-101905 online supplemental figure 1

10.1136/gpsych-2024-101905 online supplemental file 1
