# Supplementary figures and images for: Decline in activities of daily living in the rarer dementias
Source: Gen Psychiatr. 2025 Jun 8;38(3):e101905. doi: 10.1136/gpsych-2024-101905 (PMC12161298; doi:10.1136/gpsych-2024-101905)

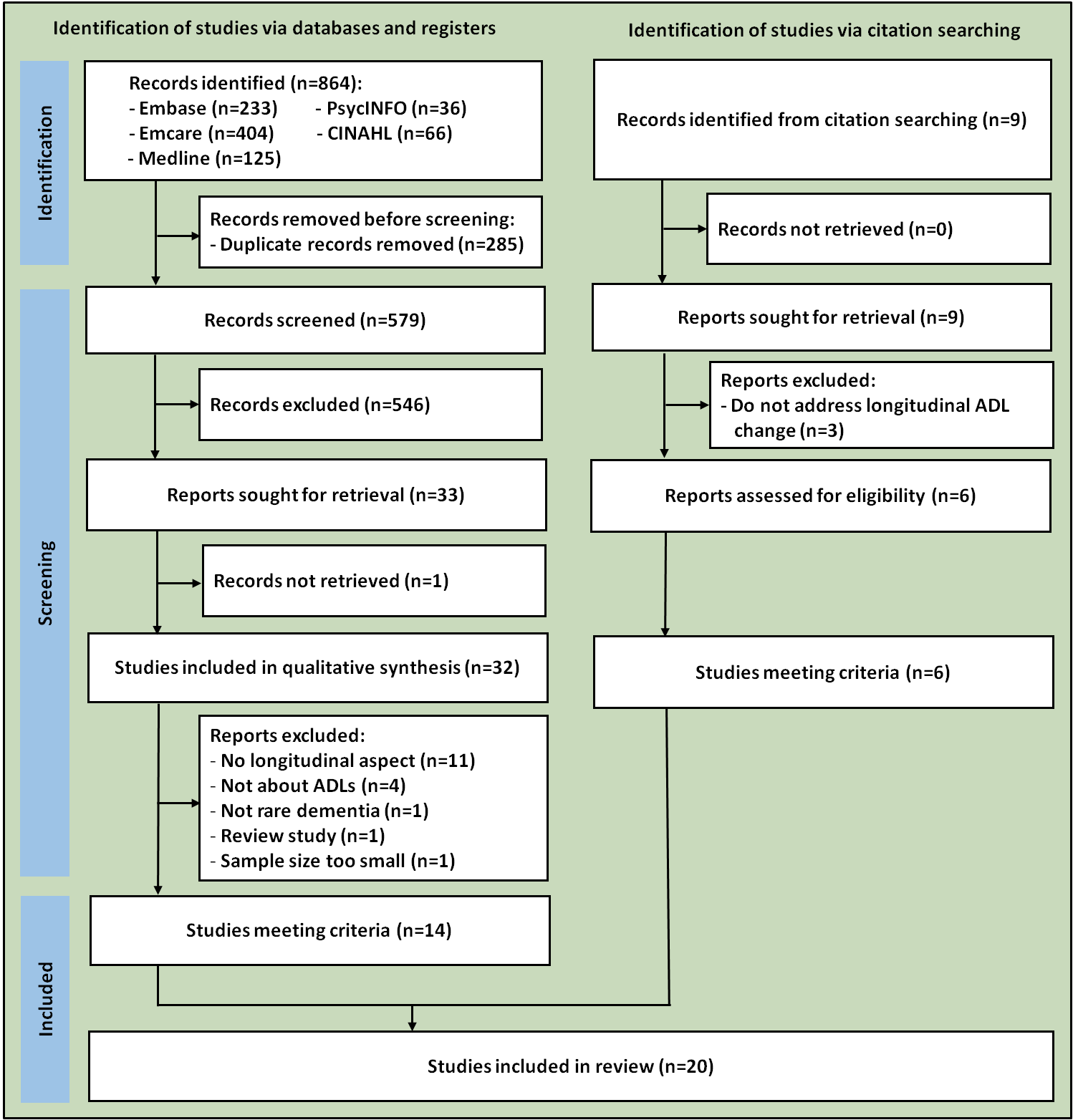

Supplement: online supplemental figure 1 [file gpsych-38-3-s001.tif]
