# Supplementary material for: Decline in activities of daily living in the rarer dementias
Source: Gen Psychiatr. 2025 Jun 8;38(3):e101905. doi: 10.1136/gpsych-2024-101905 (PMC12161298; doi:10.1136/gpsych-2024-101905)
Supplement: online supplemental file 1 [file gpsych-38-3-s002.docx]

# **SUPPLEMENTARY MATERIALS**

## **Search strategy**

A search was carried out in Medline, Embase, Emcare, PsychINFO and Cinahl in September 2021. The searches are given below.

Medline

1. Epidemiologic studies/ OR exp Case control studies/ OR exp Cohort studies/ OR case control.tw. OR (cohort adj (study OR studies)).tw. OR cohort analy$.tw. OR (follow up adj (study OR studies)).tw. OR (observational adj (study OR studies)).tw. OR longitudinal.tw. OR retrospective.tw. OR cross sectional.tw. OR cross-sectional studies/
2. primary progressive aphasia$.tw. OR exp Aphasia, primary progressive/ OR (semantic dementia OR svPPA).tw. OR (logopenic aphasia OR lvPPA).tw. OR (progressive nonfluent aphasia OR nfvPPA).tw. OR (posterior cortical atrophy OR bensons syndrome).tw. OR frontotemporal dementia.tw. OR exp Frontotemporal Lobar Degeneration/ OR (frontotemporal lobar degeneration OR frontotemporal degeneration OR Picks disease).tw. OR fFTD.tw. OR familial frontotemporal dementia.tw. OR (behavioral variant frontotemporal dementia OR behavioural variant frontotemporal dementia).tw. OR familial alzheimers disease.tw. OR atypical alzheimers disease.tw. OR frontal variant alzheimers disease.tw. OR rare dementia$.tw.
3. (carer$ OR "care need$" OR "care partner$" OR caregiver$).mp. OR Caregivers/ OR "Activities of Daily Living"/ OR activities of daily living.tw. OR (IADLs OR IADL).tw. OR (ADLs OR ADL).tw.
4. 1 AND 2 AND 3 limited to English

Embase

1. Clinical study/ OR exp case control study/ OR Family study/ OR Longitudinal study/ OR Retrospective study/ OR (Prospective study/ NOT Randomized controlled trials/) OR Cohort analysis/ OR (Cohort adj (study OR studies)).mp. OR (Case control adj (study OR studies)).tw. OR (follow up adj (study OR studies)).tw. OR (observational adj (study OR studies)).tw. OR (epidemiologic$ adj (study OR studies)).tw. OR (cross sectional adj (study OR studies)).tw.
2. primary progressive aphasia$.tw. OR exp primary progressive aphasia/ OR (semantic dementia OR svPPA).tw. OR (logopenic aphasia OR lvPPA).tw. OR (progressive nonfluent aphasia OR nfvPPA).tw. OR (posterior cortical atrophy OR bensons syndrome).tw. OR frontotemporal dementia.tw. OR exp frontotemporal dementia/ OR (frontotemporal degeneration OR frontotemporal lobar degeneration OR Picks disease).tw. OR (behavioural variant frontotemporal dementia OR behavioral variant frontotemporal dementia).tw. OR (familial frontotemporal dementia OR fFTD).tw. OR familial alzheimers disease.tw. OR atypical alzheimers disease.tw. OR frontal variant alzheimers disease.tw. OR rare dementia$.tw.
3. (carer$ OR "care needs" OR "care partner$" OR caregiver$).mp. OR Caregivers/ OR daily life activity/ OR activities of daily living.tw. OR (IADLs OR IADL).tw. OR (ADLs OR ADL).tw.
4. 1 AND 2 AND 3 limited to English

Emcare

1. exp Clinical study/ OR exp case control study/ OR Family study/ OR Longitudinal study/ OR Retrospective study/ OR (Prospective study/ NOT Randomized controlled trials/) OR
   Cohort analysis/ OR (Cohort adj (study OR studies)).mp. OR (Case control adj (study OR studies)).tw. OR (follow up adj (study OR studies)).tw. OR (observational adj (study OR studies)).tw. OR (epidemiologic$ adj (study OR studies)).tw. OR (cross sectional adj (study OR studies)).tw.
2. primary progressive aphasia$.tw. OR exp primary progressive aphasia/ OR (semantic dementia OR svPPA).tw. OR (logopenic aphasia OR lvPPA).tw. OR (progressive nonfluent aphasia OR nfvPPA).tw. OR (posterior cortical atrophy OR bensons syndrome).tw. OR exp brain cortex atrophy/ OR frontotemporal dementia.tw. OR exp frontotemporal dementia/ OR (frontotemporal degeneration OR frontotemporal lobar degeneration OR Picks disease).tw. OR (behavioural variant frontotemporal dementia OR behavioral variant frontotemporal dementia).tw. OR (familial frontotemporal dementia OR fFTD).tw.
   familial alzheimers disease.tw. OR atypical alzheimers disease.tw. OR frontal variant alzheimers disease.tw. OR rare dementia$.tw.
3. (carer$ OR "care needs" OR "care partner$" OR caregiver$).mp. OR Caregivers/
   daily life activity/ OR activities of daily living.tw. OR (ADL OR ADLs).tw. OR (IADLs OR IADL).tw.
4. 1 AND 2 AND 3 limited to English

PsycINFO

1. Cohort analysis/ OR (Cohort adj (study OR studies)).mp. OR (Case control adj (study OR studies)).tw. OR (follow up adj (study OR studies)).tw. OR (observational adj (study OR studies)).tw. OR (epidemiologic$ adj (study OR studies)).tw. OR (cross sectional adj (study OR studies)).tw. OR longitudinal.tw. OR exp retrospective studies/
   exp longitudinal studies/ OR followup studies/ OR
2. primary progressive aphasia$.tw. OR (semantic dementia OR svPPA).tw. OR (logopenic aphasia OR lvPPA).tw. OR (progressive nonfluent aphasia OR nfvPPA).tw. OR exp semantic dementia/ OR (posterior cortical atrophy OR bensons syndrome).tw. OR frontotemporal dementia.tw. OR (frontotemporal lobar degeneration OR frontotemporal degeneration OR Picks disease).tw. OR fFTD.tw. OR familial frontotemporal dementia.tw. OR (behavioral variant frontotemporal dementia OR behavioural variant frontotemporal dementia).tw. OR familial alzheimers disease.tw. OR atypical alzheimers disease.tw. OR frontal variant alzheimers disease.tw. OR rare dementia$.tw.
3. (carer$ OR "care need$" OR "care partner$" OR caregiver$).mp. OR caregivers/ OR exp "Activities of Daily Living"/ OR activities of daily living.tw. OR (IADLs OR IADL).tw. OR (ADLs OR ADL).tw.
4. 1 AND 2 AND 3 limited to English

Cinahl

1. IADLs OR IADL OR ADLs OR ADL OR "activities of daily living" OR (MH "Altered Activities of Daily Living (NANDA)") OR (MH "Self Care: Activities of Daily Living (Iowa NOC)") OR (MH "Self-Care: Instrumental Activities of Daily Living (Iowa NOC)") OR (MH "Activities of Daily Living (Saba CCC)") OR (MH "Instrumental Activities of Daily Living Alteration (Saba CCC)") OR (MH "Instrumental Activities of Daily Living (Saba CCC)") OR (MH "Activities of Daily Living (Saba CCC)") OR (MH "Activities of Daily Living") OR (MH "Caregivers") OR carer* OR "care need*" OR "care partner*" OR "caregiver*"
2. "rare dementia*" OR "frontal variant alzheimers disease" OR "atypical alzheimers disease" OR "behavioural variant frontotemporal dementia" OR "behavioral variant frontotemporal dementia" OR "frontotemporal lobar degeneration" OR "frontotemporal degeneration" OR "picks disease" OR "primary progressive aphasia" OR nfvPPA OR "semantic dementia" OR svPPA OR "logopenic dementia" OR lvPPA OR "familial alzheimer's disease" OR "familial frontotemporal dementia" OR fFTD OR (MH "Frontotemporal Dementia+") OR "frontotemporal dementia" OR "posterior cortical atrophy" OR "bensons syndrome" OR "primary progressive aphasia"
3. "observational study" OR "observational studies" OR "cohort study" OR "cohort studies" OR (MH "Cross sectional studies") OR (MH "Nonconcurrent prospective studies") OR (MH "Correlational studies") OR (MH "Case Control Studies+") OR (MH "Prospective studies")
4. 1 AND 2 AND 3 limited to English

## **Study selection**

In total 32 papers reached the final stage of the filtering process, which were then included or excluded based on whether they met every point of the criteria. For example *Diehl‐Schmid J, Richard‐Devantoy S, Grimmer T, Förstl H, Jox R. Behavioral variant frontotemporal dementia: advanced disease stages and death. A step to palliative care. International journal of geriatric psychiatry. 2017 Aug;32(8):876-81.* was excluded at this point, as whilst it addressed ADLs in a rarer dementia, it was not longitudinal. Other studies failed to meet the criteria as they focussed on psychosocial domains rather than ADLs, for example *Bak TH, Crawford LM, Berrios G, Hodges JR. Behavioural symptoms in progressive supranuclear palsy and frontotemporal dementia. Journal of Neurology, Neurosurgery & Psychiatry. 2010 Sep 1;81(9):1057-9.*

**Supplementary Figure 1: Flow diagram for literature search and selection, adapted from PRISMA**

| **Study** | **Diagnosis of Participants in study** | **Contemporary Diagnosis of Participants in study** |
| --- | --- | --- |
| Ahmed et al. 2020 [1] | PCA | PCA |
|  | AD | AD |
| Binetti et al. 2000 [2] | Pick’s Disease (PcD) | FTD |
|  | AD | AD |
| Ferrari et al (2019) [3] | lvPPA | lvPPA |
|  | nfvPPA | nfvPPA |
|  | svPPA | svPPA |
| Foxe et al (2021) [4] | lvPPA | lvPPA |
|  | nfvPPA | nfvPPA |
|  | svPPA | svPPA |
| Giebel et al (2021) [5] | bvFTD | bvFTD |
|  | AD | AD |
| Ikeda et al (2002) [6] | Frontal variant FTD (fv-FTD) | bvFTD |
|  | Semantic dementia (SD) | svPPA |
|  | AD | AD |
| Jang et al (2012) [7] | Progressive non-fluent aphasia (PNFA) | nfvPPA |
|  | Logopenic progressive aphasia (LPA) | lvPPA |
|  | AD | AD |
| Kashibayashi et al (2010) [8] | Semantic dementia (SD) | svPPA |
| Le Rhun et al (2005) [9] | PPA | PPA |
| Lima-Silva et al (2021) [10] | bvFTD | bvFTD |
|  | PPA | PPA |
|  | AD | AD |
| Mioshi et al (2009) [11] | bvFTD | bvFTD |
|  | Semantic dementia (SemDem) | svPPA |
|  | Progressive non-fluent aphasia (PNFA) | nfvPPA |
| Mioshi et al (2010) [12] | bvFTD | bvFTD |
|  | Progressive non-fluent aphasia (PNFA) | nfvPPA |
|  | Semantic dementia (SemD) | svPPA |
| Mioshi et al (2007) [13] | Progressive non-fluent aphasia (PNFA) | nfvPPA |
|  | Semantic dementia | svPPA |
|  | bvFTD | bvFTD |
|  | AD | AD |
| Moeller et al (2021) [14] | PPA | PPA |
| Morrow et al (2021) [15] | PPA | PPA |
| O’Connor et al (2016a) [16] | svPPA | svPPA |
|  | nfvPPA | nfvPPA ] |
| O’Connor et al (2016b) [17] | bvFTD | bvFTD |
|  | svPPA | svPPA |
| Pasquier et al (1999) [18] | FTD | FTD |
| Rascovsky et al (2005) [19] | FTD | FTD |
|  | AD | AD |
| Yassuda et al (2018) [20] | bvFTD | bvFTD |

Supplementary Table 1: We coded frontal variant FTD (fv-FTD) as bvFTD since the study identified it as the frontotemporal dementia variant with reported behavioural changes [37]. The terminology used reflects changing diagnostic criteria; Pick’s disease is now used to refer to the pathological presence of Pick’s bodies which can only be confirmed at autopsy.

PCA, Posterior Cortical Atrophy; AD, Alzheimer’s Disease; PPA, Primary Progressive Aphasia; lvPPA, logopenic variant PPA; nfvPPA, non-fluent/agrammatic variant PPA; PNFA, progressive non-fluent aphasia; svPPA, semantic variant PPA; FTD, Frontotemporal Dementia; bvFTD, behavioural variant FTD; fvFTD, frontal variant FTD.

PCA, Posterior Cortical Atrophy; AD, Alzheimer’s Disease; PcD, Pick’s Disease; PPA, Primary Progressive Aphasia; PPA A$\beta$-, Amyloid beta negative PPA; PPA A$\beta$+, Amyloid beta positive PPA; lvPPA, logopenic variant PPA; nfvPPA, non-fluent/agrammatic variant PPA; PNFA, progressive non-fluent aphasia; svPPA, semantic variant PPA; SD, semantic dementia; FTD, Frontotemporal Dementia; bvFTD, behavioural variant FTD; fvFTD, frontal variant FTD, PSEN1, presenilin-1; PGRN, progranulin.

| **Study** | **Design** | **Participants** | **Diagnostic criteria** | **Setting** | **ADL measure** | **Analysis method** | **Main outcomes of interest** |
| --- | --- | --- | --- | --- | --- | --- | --- |
| Ahmed et al. 2020 [1] | Cross-sectional | PCA (n=29) | Clinical. | Oxford Cognitive Disorders Clinic, UK | Disability Assessment for Dementia (DAD) | Statistical and linear regression | In PCA, management of finances, correspondence and meal preparation were impaired first. Later in the disease course, there was comparable impairment across IADLs and BADLs. BADLs were significantly more impaired in PCA vs AD (p<0.05). |
|  |  | AD (n=25) |  | Addenbrookes Early Onset Dementia Clinic, UK |  |  |  |
| Binetti et al. 2000 [2] | Cohort | Pick’s disease (PcD) (n=44) | Clinical.  26 (10 PcD, 16 AD) had pathological confirmation | Memory Disorders unit- Massachusetts general hospital, US | Blessed Dementia Scale (BDS),  Weintraub Activities of Daily Living Scale | Statistical and linear regression | Rate of decline was significantly faster in Pick’s disease than AD according to the BDS and Weintraub ADL. No Pick’s disease carers reported ADL impairments as the first symptom. |
|  |  | AD (n=121) |  |  |  |  |  |
|  |  | Controls (n=60) |  | Living in the community |  |  |  |
| Ferrari et al. 2019 [3] | Case series | lvPPA (n=22 sporadic; n=1 PSEN1 mutation) | Clinical and neuroimaging. | Neurology department at Careggi hospital, Italy | The Katz Index of Independence in Activities of Daily Living (Katz ADL) | Statistical and logistic regression | Across variants, 14 people were severely dependent in BADLs 2.57 (mean) years from baseline. Of the three subjects with a genetic mutation, one had impaired BADLs after two years. |
|  |  | nfvPPA (n=25 sporadic; n=1 PGRN mutation) |  |  |  |  |  |
|  |  | svPPA (n=18 sporadic; n=1 PGRN mutation) |  |  |  |  |  |
| Foxe et al. 2021 [4] | Cohort | lvPPA (n=41) | Clinical and neuroimaging | FRONTIER Frontotemporal Dementia Group, Australia | Disability Assessment for Dementia (DAD) | Statistical, mixed and hierarchal regression modelling | Annual rate of decline on DAD comparable across variants, (8.7 points for lvPPA and nfvPPA, 7.4 points for svPPA). |
|  |  | nfvPPA (n=44) |  |  |  |  |  |
|  |  | svPPA (n=62) |  |  |  |  |  |
|  |  | Controls (n=60) |  |  |  |  |  |
| Giebel et al. 2021  [5] | Cohort | bvFTD (n=306) | Clinical | National Alzheimer’s Coordinating Center (NACC), US | Functional Activities Questionnaire (FAQ) | Statistical and linear mixed effects model | bvFTD was significantly more impaired longitudinally than tAD (p<0.001). Rates of change was comparable in bvFTD and tAD, except for ‘using the stove’ (p<0.01) and in ‘travel’ (p<0.05) which declined more rapidly in bvFTD. |
|  |  | AD (n=3045) |  |  |  |  |  |
| Ikeda et al. 2002 [6] | Cohort | fvFTD (n=23) | Clinical | Cognitive Disorders Clinic, Cambridge, UK | Custom informant questionnaire | Statistical | In SD, food preference changed first, followed by increased appetite, altered eating habits, other oral behaviours, and finally swallowing issues. In fvFTD, altered eating habits and increased appetite were the initial symptoms. |
|  |  | SD (n=25) |  |  |  |  |  |
|  |  | AD (n=43) |  |  |  |  |  |
| Jang et al. 2012 [7] | Cross-sectional | PNFA (n=16) | Clinical | FRONTIER Frontotemporal Dementia Group , Australia | Disability Assessment for Dementia (DAD) | Statistical | All 3 groups declined in IADLs over 12 months, but only nfvPPA declined in BADLs over the same period (p<0.05).  Both nfvPPA and lvPPA showed decline in planning and execution at 12 months. |
|  |  | LPA (n=19) |  |  |  |  |  |
|  |  | AD (n=24) |  |  |  |  |  |
| Kashibayashi et al. 2010 [8] | Case series | svPPA (n=19) | Clinical | Higher Brain Function Clinic, Ehime, Japan | Custom informant questionnaire | Statistical | ADL decline followed a specific pattern: reading/writing, efficiency of work/housework, daily activities, eating, continence, and dressing. 14 patients showed a decline in ADLs after an average 5.4 years from onset. Five years after onset, some patients needed specialized care due to eating disturbances, dressing impairments, and incontinence. |
| Rhun et al. 2005 [9] | Case series | PPA (n=49) | Clinical | Lille Memory Centre, France | Custom informant questionnaire | Statistical | Half the patients required assistance in toileting, hygiene or dressing 5 years from onset. Half required help eating and walking 7-8 years from onset. |
| Lima-Silva et al. 2021 [10] | Cohort | bvFTD (n=31) | Clinical | Cognitive and Behavioral Neurology Group, University of Sao Paulo; Cognitive and Behavioral Neurology Group, Federal University of Minas Gerais; Department of Neurology, State University of Campinas | FTD Rating Scale (FRS),  Clinical Dementia Scale-frontotemporal lobar degeneration (CDR‐FTLD), Clinical Dementia Scale (CDR) | Statistical | Greater decline at 12 months in bvFTD and PPA, than in AD. The FTD Rating Scale did not detect significant decline in PPA (p=0.69). The Clinical Dementia Scale was less sensitive to severe disease stages. |
|  |  | PPA (n=12) |  |  |  |  |  |
|  |  | AD (n=27) |  |  |  |  |  |
| Mioshi et al. 2009 [11] | Case series | bvFTD phenocopy (n=10) | Clinical and neuroimaging. Phenocopy classified as lack of atrophy on MRI at baseline and follow-up. | Unclear | Disability Assessment for Dementia (DAD) | Statistical | In non-pathological bvFTD, there was no significant change observed. In the pathologically verified bvFTD cases they found similar decline across initiation, planning and execution at 12 month follow up. SD and PNFA declined in planning at 12 months; PNFA additionally declined in initiation. |
|  |  | bvFTD pathology (n=6) |  |  |  |  |  |
|  |  | SD (n=11) |  |  |  |  |  |
|  |  | PNFA (n=9) |  |  |  |  |  |
| Mioshi et al. 2010 [12] | Cohort | bvFTD (n=57) | Clinical and neuroimaging | Cambridge, UK | FTD Rating Scale (FRS), Clinical Dementia Scale (CDR) | Statistical | All variants had significant decline on the FTD Rating Scale (p<0.005) at 12 months. Individuals with bvFTD had greater functional loss than those with SD or PNFA. |
|  |  | PNFA (n=41) |  |  |  |  |  |
|  |  | SD (n=54) |  |  |  |  |  |
|  |  | Controls (n=20) |  |  |  |  |  |
| Mioshi et al. 2007 [13] | Cross-Sectional | PNFA (n=10) | Clinical and neuroimaging | Addenbrookes Hospital Early Onset Dementia Clinic, UK | Disability Assessment for Dementia (DAD),  Clinical Dementia Scale (CDR) | Statistical | At 12 months, PNFA showed little decline in BADLs but impairment in language-related IADLs like using the telephone and managing finance/correspondence. SD group had broader IADL impairment; most remained independent but some struggled with weather-appropriate clothing. Outings and leisure/house chores were mildly impaired. bvFTD group experienced the most severe impairments in both BADLs and IADLs, particularly in continence, dressing, eating and hygiene. Management of finances/correspondence and outings were the worst affected IADLs. |
|  |  | SD (n=15) |  |  |  |  |  |
|  |  | bvFTD (n=15) |  |  |  |  |  |
|  |  | AD (n=19) |  |  |  |  |  |
| Moeller et al. 2021 [14] | Cohort | PPA A$\beta$- (n=11) | Clinical and Pathological | Mesulam Center for Cognitive Neurology and Alzheimer’s Disease, US | Activities of Daily Living Questionnaire (ADLQ) | Statistical and linear mixed effects model | The A$\beta$+ group were worse at baseline and declined faster on the Activities of Daily Living Questionnaire. No symptom sequence was explicitly described but from the results we infer that PPA A$\beta$+ declined as follows: communication, employment and recreation, shopping and money, travel, household care and finally self-care. The PPA A$\beta-$ group declined in a similar sequence: communication, employment and recreation, travel, household care, shopping and money, and self-care. |
|  |  | PPA A$\beta$+ (n=17) |  |  |  |  |  |
| Morrow et al. 2021 [15] | Case series | PPA (n=1944). 18% were nfvPPA, 12% were SD, remainder were not designated a variant. Results were not split by variant. | Clinical | National Alzheimer’s Coordinating Center (NACC), US | Functional Activities Questionnaire (FAQ),  National Alzheimer’s Disease Coordinating Centre Frontotemporal Lobar Degeneration (NACC-FTLD) module, Clinical Dementia Scale (CDR) | Statistical | The sequence of functional decline was: verbal communication, transactions, meal preparation, self-care and ambulation. |
| O’Connor et al. 2016a [16] | Case series | svPPA (n=18) | Clinical | FRONTIER Frontotemporal Dementia Group , Australia | Disability Assessment for Dementia (DAD) | Statistical and linear regression | The nfvPPA group experienced more significant IADL decline at the 12-month follow-up. |
|  |  | nfvPPA (n=11) |  |  |  |  |  |
| O’Connor et al. 2016  [17] | Cohort | bvFTD (n=21) | Clinical | FRONTIER Frontotemporal Dementia Group , Australia | Disability Assessment for Dementia (DAD) | Statistical | In bvFTD, the initial ADL decline was characterized by overeating, while in SD, it manifested as rigid routines and unusual food preferences. |
|  |  | SD (n=18) |  |  |  |  |  |
| Pasquier et al. 1999 [18] | Case series | FTD (n=74) | Clinical and neuroimaging | Lille Memory Clinic, France | Weintraub Activities of Daily Living Scale | No statistical analysis | After two years there was a median 10% loss of autonomy. In the final stages eating and dressing were the best-preserved activities. |
| Rascovsky et al. 2005 [19] | Cohort | FTD (n=70) | Clinical and autopsy confirmation | National Alzheimer’s Coordinating Center (NACC), US | Custom informant questionnaire | Statistical | FTD showed a quicker decline in BADLs. Specifically, compared to AD, FTD lost capacity for bathing, dressing, grooming, and toileting at a faster rate (p<0.01). |
|  |  | AD (n=70) |  |  |  |  |  |
| Yassuda et al. 2018 [20] | Cross-sectional | bvFTD (n=109) | Clinical and neuroimaging | FRONTIER Frontotemporal Dementia Group , Australia; Cognitive and Behavioral Neurology Group, University of Sao Paulo; Cognitive and Behavioral Neurology Group, Federal University of Minas Gerais; Department of Neurology, State University of Campinas  Brazil, Cognitive and Behavioral; Addenbrookes Early onset dementia clinic, UK; Nizam’s Institute of Medical Sciences, India | Disability Assessment for Dementia (DAD) | Statistical and linear regression | Cross-sectionally compared groups according to DAD, but no results on rate or sequence of ADL decline. |

*Supplementary Table 2:* Main outcomes of interest for the 20 studies included in the review.

## **Quality assessment**

| **Quality Assessment** |  |  |  |  |  |  |  |  |  |  |
| --- | --- | --- | --- | --- | --- | --- | --- | --- | --- | --- |
|  | Ahmed et al (2020) [1] | Binetti et al (2000) [2] | Ferrari et al (2019) [3] | Foxe et al (2021) [4] | Giebel et al (2021) [5] | Ikeda et al (2002) [6] | Jang et al (2012) [7] | Kashibayashi et al (2010) [8] | Le Rhun et al (2005) [9] | Lima-Silva et al (2021) [10] |
| Were the groups similar and recruited from the same population? | Yes | Yes | Yes | Yes | Yes | Yes | Yes | NA | NA | Yes |
| Was the setting described in detail? | Yes | Yes | Yes | Yes | Yes | Yes | Yes | Yes | Yes | Yes |
| Were the criteria for inclusion in the sample clearly defined? | Yes | Yes | Yes | Yes | Yes | Yes | Yes | Yes | Yes | Yes |
| Were objective, standard criteria used for diagnosis? | Yes | Yes | Yes | Yes | Yes | Yes | Yes | Yes | Yes | Yes |
| Was there clear reporting of the demographics of the participants in the study? | Yes | Yes | Yes | Yes | Yes | Yes | Yes | Yes | Yes | Yes |
| Were confounding factors identified? | Yes | Yes | Yes | Yes | Yes | Yes | Yes | No | Yes | Yes |
| Were strategies to deal with confounding factors stated? | Yes | Yes | Yes | Yes | Yes | Yes | Yes | No | Yes | Yes |
| Were ADLs measured in a valid and reliable way? | Yes | Yes | Yes | Yes | Yes | Yes | Yes | Yes | Yes | Yes |
| Was the timescale (longitudinal/cross-sectional) sufficient for outcomes to occur? | Yes | Yes | Yes | Yes | Yes | Yes | Yes | Yes | Yes | Yes |
| Was follow up complete? | NA | No | Yes | No | No | NA | Yes | No | Yes | No |
| Were the reasons for loss to follow up described? | NA | Yes | NA | No | Yes | NA | NA | Yes | NA | No |
| Were the outcomes or follow up results of cases clearly reported? | Yes | Yes | Yes | Yes | Yes | Yes | Yes | Yes | Yes | Yes |
| Were conclusions clearly supported by the data? | Yes | Yes | Yes | Yes | Yes | Yes | Yes | Yes | Yes | Yes |
| Was the relevance and transferability evident? | Yes | Yes | Yes | Yes | Yes | Yes | Yes | Yes | Yes | Yes |
| Was statistical analysis appropriate? | Yes | Yes | Yes | Yes | Yes | Yes | Yes | Yes | Yes | Yes |
| Total | 13/13 | 14/15 | 14/14 | 13/15 | 14/15 | 13/13 | 14/14 | 11/14 | 13/13 | 13/15 |

| **Quality Assessment** |  |  |  |  |  |  |  |  |  |  |
| --- | --- | --- | --- | --- | --- | --- | --- | --- | --- | --- |
|  | Mioshi et al (2009) [11] | Mioshi et al (2010) [12] | Mioshi et al (2007) [13] | Moeller et al (2021) [14] | Morrow et al (2021) [15] | O’Connor et al (2016a) [16] | O’Connor et al (2016b) [17] | Pasquier et al (1999) [18] | Rascovsky et al (2005) [19] | Yassuda et al (2018) [20] |
| Were the groups similar and recruited from the same population? | Unclear | Yes | Yes | Yes | NA | Yes | Yes | NA | Yes | NA |
| Was the setting described in detail? | No | Yes | Yes | Yes | Yes | Yes | Yes | Yes | Yes | Yes |
| Were the criteria for inclusion in the sample clearly defined? | Yes | Yes | Yes | Yes | Yes | Yes | Yes | Yes | Yes | Yes |
| Were objective, standard criteria used for diagnosis? | Yes | Yes | Yes | Yes | Unclear | Yes | Yes | Yes | Yes | Yes |
| Was there clear reporting of the demographics of the participants in the study? | Yes | Yes | Yes | Yes | Yes | Yes | Yes | Yes | Yes | Yes |
| Were confounding factors identified? | Yes | Yes | Yes | Yes | Yes | Yes | Yes | No | No | Yes |
| Were strategies to deal with confounding factors stated? | Yes | Yes | Yes | Yes | Yes | Yes | Yes | No | No | Yes |
| Were ADLs measured in a valid and reliable way? | Yes | Yes | Yes | Yes | Yes | Yes | Yes | Yes | Yes | Yes |
| Was the timescale (longitudinal/cross-sectional) sufficient for outcomes to occur? | Yes | Yes | Yes | Yes | Yes | Yes | Yes | Yes | Yes | Yes |
| Was follow up complete? | Yes | No | NA | No | No | No | No | No | No | NA |
| Were the reasons for loss to follow up described? | NA | No | NA | Yes | No | No | Yes | Yes | Yes | NA |
| Were the outcomes or follow up results of cases clearly reported? | Yes | Yes | Yes | Yes | Yes | Yes | Yes | Yes | Yes | Yes |
| Were conclusions clearly supported by the data? | Yes | Yes | Yes | Yes | Yes | Yes | Yes | Yes | Yes | Yes |
| Was the relevance and transferability evident? | Yes | Yes | Yes | Yes | Yes | Yes | Yes | Yes | Yes | Yes |
| Was statistical analysis appropriate? | Yes | Yes | Yes | Yes | Yes | Yes | Yes | No | Yes | Yes |
| Total | 12/14 | 13/15 | 13/13 | 14/15 | 11/14 | 13/15 | 14/15 | 11/14 | 12/15 | 12/12 |

Supplementary Table 3: Custom quality assessment of included studies. Each column represents an individual study; rows represent individual quality criterion. The final ‘Total’ row gives the total number of criteria met by each study.

| **COHORT** | Author (year) | | | | | |
| --- | --- | --- | --- | --- | --- | --- |
|  | Binetti et al (2000) [2] | Foxe et al (2021) [4] | Giebel et al (2021) [5] | Jang et al. 2012 [7] | Rascovsky et al (2005) [19] | Lima-Silva et al (2021) [10] |
| Were the two groups similar and recruited from the same population? | Yes | Yes | Yes | Yes | Yes | Yes |
| Were the exposures measured similarly to assign people to both exposed and unexposed groups? | Yes | Yes | Yes | Yes | Yes | Yes |
| Was the exposure measured in a valid and reliable way? | Yes | Yes | Yes | Yes | Yes | Yes |
| Were confounding factors identified? | Yes | Yes | Yes | Yes | No | Yes |
| Were strategies to deal with confounding factors stated? | Yes | Yes | Yes | Yes | No | Yes |
| Were the groups/participants free of the outcome at the start of the study (or at the moment of exposure)? | Yes | Yes | Yes | Yes | Yes | Yes |
| Were the outcomes measured in a valid and reliable way? | Yes | Yes | Yes | Yes | Yes | Yes |
| Was the follow up time reported and sufficient to be long enough for outcomes to occur? | Yes | Yes | Yes | Yes | Yes | Yes |
| Was follow up complete, and if not, were the reasons to loss to follow up described and explored? | No | No | Yes | No | Yes | Yes |
| Were strategies to address incomplete follow up utilized? | No | No | No | No | No | NA |
| Was appropriate statistical analysis used? | Yes | Yes | Yes | Yes | Yes | Yes |
| Total (/11) | 9 | 9 | 10 | 9 | 8 | 10 |

Supplementary Table 4: Joanna Briggs Institute critical assessment of cohort studies. Each column represents an individual study; rows represent individual quality criterion. The final ‘Total’ row gives the total number of criteria met by each study.

| **CROSS-SECTIONAL** | Author (year) | | | |
| --- | --- | --- | --- | --- |
|  | Ahmed et al. 2020 [1] | Ikeda et al (2002) [6] | Mioshi et al (2007) [13] | Yassuda et al. 2018 [20] |
| Were the criteria for inclusion in the sample clearly defined? | Yes | Yes | Yes | Yes |
| Were the study subjects and the setting described in detail? | Yes | Yes | Yes | Yes |
| Was the exposure measured in a valid and reliable way? | Yes | Yes | Yes | Yes |
| Were objective, standard criteria used for measurement of the condition? | Yes | Yes | Yes | Yes |
| Were confounding factors identified? | Yes | Yes | Yes | Yes |
| Were strategies to deal with confounding factors stated? | Yes | Yes | Yes | Yes |
| Were the outcomes measured in a valid and reliable way? | Yes | Yes | Yes | Yes |
| Was appropriate statistical analysis used? | Yes | Yes | Yes | Yes |
| Total (/8) | 8 | 8 | 8 | 8 |

Supplementary Table 5: Joanna Briggs Institute critical assessment of cross-sectional studies. Each column represents an individual study; rows represent individual quality criterion. The final ‘Total’ row gives the total number of criteria met by each study.

| **CASE SERIES** | Author (year) | | | | | | | | | |
| --- | --- | --- | --- | --- | --- | --- | --- | --- | --- | --- |
|  | Ferrari et al. 2019 [3] | Kashibayashi et al (2010) [8] | Le Rhun et al (2005) [9] | Mioshi et al (2009) [11] | Mioshi et al (2010) [12] | Pasquier et al (1999) [18] | Morrow et al (2021) [15] | O’Connor et al (2016a) [16] | O’Connor et al (2016b) [17] | Pasquier et al (1999) [18] |
| Were there clear criteria for inclusion in the case series? | Yes | Yes | Yes | Yes | Yes | Yes | Yes | Yes | Yes | Yes |
| Was the condition measured in a standard, reliable way for all participants included in the case series? | Yes | Yes | Yes | Yes | Yes | Yes | Yes | Yes | Yes | Yes |
| Were valid methods used for identification of the condition for all participants included in the case series? | Yes | Yes | Yes | Yes | Yes | Yes | Yes | Yes | Yes | Yes |
| Did the case series have consecutive inclusion of participants? | Unclear | Yes | Yes | No | Yes | Unclear | Unclear | Yes | Unclear | Yes |
| Did the case series have complete inclusion of participants? | No | No | Yes | No | No | Unclear | Unclear | No | No | Yes |
| Was there clear reporting of the demographics of the participants in the study? | Yes | Yes | Yes | Yes | Yes | Yes | Yes | Yes | Yes | Yes |
| Was there clear reporting of clinical information of the participants? | Yes | Yes | Yes | Yes | Yes | Yes | Yes | Yes | Yes | Yes |
| Were the outcomes or follow up results of cases clearly reported? | Yes | Yes | Yes | Yes | Yes | Yes | Yes | Yes | Yes | Yes |
| Was there clear reporting of the presenting site(s)/clinic(s) demographic information? | No | No | No | No | Yes | Yes | No | No | Yes | Yes |
| Was statistical analysis appropriate? | Yes | Yes | Yes | Yes | Yes | Yes | Yes | Yes | Yes | Yes |
| Total (/10) | 7 | 8 | 9 | 7 | 9 | 8 | 7 | 8 | 8 | 10 |

Supplementary Table 6: Joanna Briggs Institute critical assessment of case series studies. Each column represents an individual study; rows represent individual quality criterion. The final ‘Total’ row gives the total number of criteria met by each study.

# **Bibliography**

1 Ahmed S, Culley S, Blanco-Duque C, *et al.* Pronounced Impairment of Activities of Daily Living in Posterior Cortical Atrophy. *Dement Geriatr Cogn Disord*. 2020;49:48–55. doi: 10.1159/000506125

2 Binetti G, Locascio JJ, Corhln S, *et al.* Differences Between Pick Disease and Alzheimer Disease in Clinical Appearance and Rate of Cognitive Decline. *Arch Neurol*. 2000;57:225–32. doi: 10.1001/ARCHNEUR.57.2.225

3 Ferrari C, Polito C, Vannucchi S, *et al.* Primary Progressive Aphasia: Natural History in an Italian Cohort. *Alzheimer Dis Assoc Disord*. Published Online First: 2019.

4 Foxe D, Irish M, Hu A, *et al.* Longitudinal cognitive and functional changes in primary progressive aphasia. *J Neurol*. 2021;268:1951–61. doi: 10.1007/S00415-020-10382-9

5 Giebel CM, Knopman D, Mioshi E, *et al.* Distinguishing Frontotemporal Dementia From Alzheimer Disease Through Everyday Function Profiles: Trajectories of Change. *J Geriatr Psychiatry Neurol*. 2021;34:66–75. doi: 10.1177/0891988720901791

6 Ikeda M, Brown J, Holland AJ, *et al.* Changes in appetite, food preference, and eating habits in frontotemporal dementia and Alzheimer’s disease. *J Neurol Neurosurg Psychiatry*. 2002;73:371–6. doi: 10.1136/jnnp.73.4.371

7 Jang J, Cushing N, Clemson L, *et al.* Activities of daily living in progressive non-fluent aphasia, logopenic progressive aphasia and Alzheimer’s disease. *Dement Geriatr Cogn Disord*. 2012;33:354–60. doi: 10.1159/000339670

8 Kashibayashi T, Ikeda M, Komori K, *et al.* Transition of distinctive symptoms of semantic dementia during longitudinal clinical observation. *Dement Geriatr Cogn Disord*. 2010;29:224–32. doi: 10.1159/000269972

9 Le Rhun E, Richard F, Pasquier F. Natural history of primary progressive aphasia. *Neurology*. 2005;65:887–91. doi: 10.1212/01.WNL.0000175982.57472.84

10 Lima-Silva TB, Mioshi E, Bahia VS, *et al.* Disease Progression in Frontotemporal Dementia and Alzheimer Disease: The Contribution of Staging Scales. *J Geriatr Psychiatry Neurol*. 2021;34:397–404. doi: 10.1177/0891988720944239

11 Mioshi E, Hodges JR. Rate of change of functional abilities in frontotemporal dementia. *Dement Geriatr Cogn Disord*. 2009;28:419–26. doi: 10.1159/000255652

12 Mioshi E, Hsieh S, Savage MS, *et al.* Clinical staging and disease progression in frontotemporal dementia. 2010.

13 Mioshi E, Kipps CM, Dawson FK, *et al.* Activities of daily living in frontotemporal dementia and Alzheimer disease. 2007.

14 Moeller S, Sridhar J, Martersteck A, *et al.* Functional decline in the aphasic variant of Alzheimer’s disease. *Wiley Online Library*. 2021;17:1641–8. doi: 10.1002/alz.12331

15 Morrow CB, Leoutsakos JMS, Onyike CU. Functional Disabilities and Psychiatric Symptoms in Primary Progressive Aphasia. *The American Journal of Geriatric Psychiatry*. Published Online First: 28 July 2021. doi: 10.1016/J.JAGP.2021.07.013

16 O’Connor CM, Clemson L, Flanagan E, *et al.* The relationship between behavioural changes, cognitive symptoms, and functional disability in primary progressive aphasia: A longitudinal study. *Dement Geriatr Cogn Disord*. 2016;42:215–26. doi: http://dx.doi.org/10.1159/000449283

17 O’Connor CM, Clemson L, Hornberger M, *et al.* Longitudinal change in everyday function and behavioral symptoms in frontotemporal dementia. *AAN Enterprises*. Published Online First: 2016.

18 Pasquier F, Lebert F, Lavenu I, *et al.* The Clinical Picture of Frontotemporal Dementia: Diagnosis and Follow-Up. *Dement Geriatr Cogn Disord*. 1999;10:10–4.

19 Rascovsky K, Salmon M; DP, Lipton ; A M, *et al.* Rate of progression differs in frontotemporal dementia and Alzheimer disease. 2005.

20 Yassuda MS, Lima da Silva TB, O’Connor CM, *et al.* Apathy and functional disability in behavioral variant frontotemporal dementia. *Neurol Clin Pract*. 2018;8:120–8. doi: 10.1212/CPJ.0000000000000429

21 O’Connor CM, Clemson L, Flanagan E, *et al.* The Relationship between Behavioural Changes, Cognitive Symptoms, and Functional Disability in Primary Progressive Aphasia: A Longitudinal Study. *Dement Geriatr Cogn Disord*. 2016;42:215–26. doi: 10.1159/000449283
